# Pre-assembled ECMO: Enhancing efficiency and reducing stress in refractory cardiac arrest care

**DOI:** 10.1016/j.resplu.2024.100800

**Published:** 2024-10-18

**Authors:** Tharusan Thevathasan, Sonia Lech, Andreas Diefenbach, Elisa Bechthold, Tim Gaßmann, Sebastian Fester, Georg Girke, Wulf Knie, Benjamin T. Lukusa, Sebastian Kühn, Steffen Desch, Ulf Landmesser, Carsten Skurk

**Affiliations:** a Department of Cardiology, Angiology and Intensive Care Medicine, Deutsches Herzzentrum der Charité (DHZC), Campus Benjamin Franklin, Hindenburgdamm 30, 12203 Berlin, Germany; b Berlin Institute of Health (BIH), Charité – Universitätsmedizin Berlin, Charitéplatz 1, 10117 Berlin, Germany; c DZHK (German Centre for Cardiovascular Research), partner site Berlin, Potsdamer Str. 58, 10785 Berlin, Germany; d Department of Psychiatry and Neurosciences, Charité-Universitätsmedizin Berlin, Campus Mitte, Charitéplatz 1, 10117 Berlin, Germany; e Department of Microbiology and Infection Immunology, Charité - Universitätsmedizin Berlin, Charitéplatz 1, 10117 Berlin, Germany; f Deutsches Rheuma-Forschungszentrum (DRFZ), Virchowweg 12, 10117 Berlin, Germany; g Labor Berlin – Charité Vivantes Services GmbH, Sylter Straße 2, 13353 Berlin, Germany; h Heart Center Leipzig at the University of Leipzig, Department of Internal Medicine/Cardiology, Germany; i Helios Health Institute, Leipzig, Germany

**Keywords:** Cardiac arrest, Extracorporeal cardio-pulmonary resuscitation, Veno-arterial extracorporeal membrane oxygenation, Hygiene, Psychologic stress

## Abstract

**Aim:**

Extracorporeal cardiopulmonary resuscitation (ECPR) by veno-arterial extracorporeal membrane oxygenation (VA-ECMO) during refractory cardiac arrest presents significant medical and psychological challenges for healthcare providers. Beyond managing cardiac arrest and preparing for potential coronary angiography, the ECMO circuit must be assembled and primed under strictly sterile conditions, contributing to additional psychological stress and potential delays in ECMO cannulation. This pragmatic study thought to evaluate whether pre-assembled and pre-primed ECMO circuits (pre-primed group) maintain sterility over a 21-day period, expedite ECMO initiation in ECPR patients and alleviate the psychological burden on the ECPR team, compared to newly assembled and primed ECMO circuits (on-demand group).

**Methods:**

In a prospective manner, ECMO circuits were either pre-assembled and pre-primed under sterile conditions, maintained for 21 days with culture samples taken every seventh day, or newly assembled and primed during the acute emergency situation. The transition from on-demand assembly and priming of ECMO circuits to pre-primed ECMO circuits occurred on January 1st, 2021. The interval between patients’ arrival in the cardiac catheterization laboratory and the initiation of ECMO was recorded and retrospectively compared between the two treatment groups. The ECPR team, comprising experienced cardiologists and nurses, was prospectively surveyed using the modified Perceived Stress Questionnaire (PSQ-20).

**Results:**

All aseptically pre-assembled and pre-primed ECMO circuits demonstrated sterile cultures for aerobic and anaerobic microorganisms as well as fungal agents over the 21-day period: 0/120 positive cultures (0 %, 95 % CI for binomial probability 0–0.03). The time to ECMO initiation was significantly reduced in the pre-primed group compared to the on-demand group: 13 [IQR 9–17] versus 31 [IQR 27–44] minutes, P < 0.001. Responses from ECPR physicians and nurses on the PSQ-20 were similar across all items. With the use of pre-primed ECMO circuits, all ECPR professionals reported a greater sense of settled inner feeling, considerably less psychological tension, fewer worries and insecurities, as well as more effective ICU shifts with improved personal goal achievement. However, treating ECPR patients with pre-primed ECMO circuits did not lead to increased job satisfaction or higher physical energy levels.

**Conclusion:**

Aseptically pre-assembled and pre-primed ECMO circuits maintain sterility for multiple weeks, significantly reducing ECMO initiation times and alleviating psychological strain on the ECPR team. Consequently, implementing these circuits in ECPR centers could enhance both patient outcomes and healthcare provider well-being.

## Graphical abstract

There are four ECPR scenarios: the ECMO circuit may already be pre-assembled and pre-primed (options A and B) or require immediate assembly and priming during the emergency case (options C and D). Upon a potential ECPR candidate’s arrival, the team manages cardiac arrest care including drug and airway management, prepares for coronary angiography and handles ECMO assembly (options C and D only). If the patient regains spontaneous circulation or has contraindications for ECMO cannulation, the ECMO circuit is not used. If the patient remains in refractory cardiac arrest and meets eligibility criteria, ECPR is considered according to guidelines. Times until ECMO initiation are displayed as median and interquartile range. CPR, cardio-pulmonary resuscitation; ECMO, extracorporeal membrane oxygenation; ECPR, extracorporeal cardio-pulmonary resuscitation; IHCA, in-hospital cardiac arrest; OHCA, out-of-hospital cardiac arrest; ROSC, return of spontaneous circulation.

**Figure:**
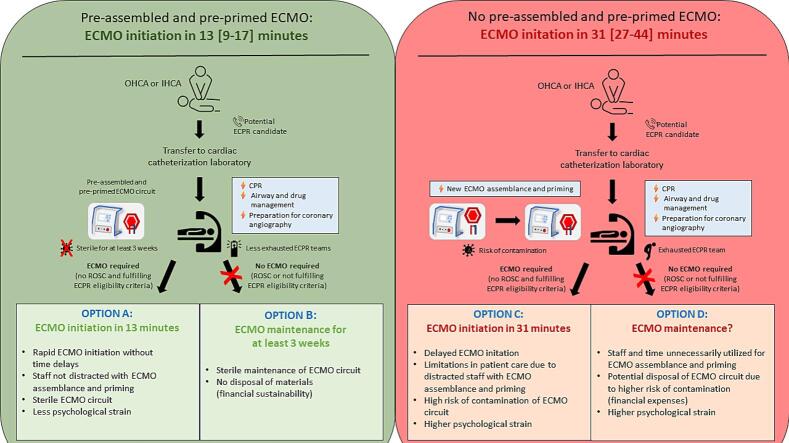

## Introduction

Cardiac arrest is the third leading cause of mortality in Europe.^1^ Extracorporeal cardiopulmonary resuscitation (ECPR) using veno-arterial extracorporeal membrane oxygenation (VA-ECMO) may be considered for cases of refractory cardiac arrest. The use of ECPR has shown the potential to improve survival and/or neurologic outcomes in recent randomized clinical trials (RCTs).^2^, ^3^, ^4^ The application of ECPR has expanded considerably in selected patients and has been endorsed in recent clinical guidelines.^5^ The duration between the onset of CPR and the initiation of VA-ECMO, known as the “low-flow time”, is a critical factor influencing patient survival. The optimal therapeutic window for ECPR initiation is suggested to be within 60 min after patient collapse.^6^

ECPR cases present medically complex and psychologically demanding situations for healthcare providers. Beyond assembling and priming the VA-ECMO circuit under strict aseptic conditions, the ECPR team is also responsible for managing cardiac arrest care and potentially preparing for coronary angiography. These additional tasks may further prolong low-flow times and heighten the psychological strain on the ECPR team. Pre-assembled and pre-primed ECMO circuits, which are maintained on “standby mode” and readily available for emergency use, have the potential to significantly reduce the time to ECMO initiation and alleviate the psychological strain on the ECPR team.

To our knowledge, this is the first study to prospectively investigate whether pre-assembled and pre-primed ECMO circuits will remain sterile for longer than 21 days, decrease the time to ECMO initiation in patients with refractory cardiac arrest and reduce the psychological strain on the ECPR team.

## Methods

This observational study was performed at a tertiary care center of the Deutsches Herzzentrum der Charité (DHZC), Berlin, Germany. The sterility testing of ECMO circuits and the anonymous ECPR team survey did not involve patients, thus an ethics protocol was not required. The Institutional Review Board approved studies measuring the time periods until ECMO initiation (protocol number: EA4/220/21). This study adheres to the STROBE guideline (Supplemental Material).

### Sterility testing of ECMO circuits

The ECMO circuits (Maquet® Cardiopulmonary GmbH, Rastatt, Germany) were prospectively assembled and primed using strict aseptic techniques by four independent and experienced members of the ECPR team. A total of 1000 mL of normal saline was used to prime each ECMO circuit. Unlike previous studies on ECMO circuit sterility,^7^ the VA-ECMO machines were not stored in a separate room restricted to research staff. This study aimed to replicate real-world clinical practice without artificial scientific conditions. Therefore, the assembled and primed ECMO circuits were covered with sterile sheets and stored in the patient recovery room of the cardiac catheterization laboratory, which is used for daily patient care. The ECMO machines were maintained in “standby mode” with a speed of 500 rotations per minute. Given the absence of comparable data in the literature, the number of culture samples was determined on an exploratory basis.

Each primed ECMO circuit was operated for 21 days, with culture samples drawn every 7th day, specifically at T_0_ (day 0), T_1_ (day 7), T_2_ (day 14), and T_3_ (day 21), resulting in four samples per ECMO circuit over the study period. At each sampling time point, study staff obtained 15 mL of fluid through the pre-pump access port of each ECMO circuit. These samples were divided equally into three vials: one aerobic, one anaerobic and one fungal culture vial (5 mL per vial). Antiseptic sprays and swabs were employed during the sampling procedures to prevent contamination of the ECMO circuit, samples and vials. The samples were then promptly delivered to the hospital's microbiology laboratory for further analysis.

At the microbiology department, samples were immediately incubated in BD BACTEC™ FX blood culture systems at 35 °C (±1.5) and agitated regularly. If metabolic activity is present during incubation, CO_2_ is released, reacting with a dye in the vial sensor of the bottle. Fluorescent substances are activated by an LED and fluorescence is modulated by the dye in the sensor. A photo detector then reads the fluorescence and the raw data are analyzed by a row board for positivity. If a blood culture turnes positive, the system flags the sample and triggers an audible alert, prompting further analysis. If a sample remains flagged as negative after incubation ended, the incubation time is verified, the results are reported and the sample is eventually disposed of.

In cases where further analysis was necessary due to a positive flag for the blood culture, the sample is processed for automated inoculation of a Columbia blood agar plate and Gram stain preparation, with immediate incubation of the blood agar plate at 36 °C (±1). Additionally, automated inoculation was performed on heated blood agar (chocolate agar), UTI chromogenic agar, sabouraud agar and schaedler agar plates, followed by their respective incubations. Growth was monitored for a minimum of three days, and further identification was carried out for any positive growth observed.

### Measurement of time to ECMO initiation

Generally, there are four ECPR scenarios (see Graphical abstract): The ECMO circuit may either be already pre-assembled and pre-primed (pre-primed group) or require immediate assembly and priming for each new potential ECPR candidate on-demand group). The transition from on-demand assembly and priming of ECMO circuits to pre-primed ECMO circuits occurred on January 1st, 2021. Upon arrival of a potential ECPR candidate in the cardiac catheterization laboratory, the ECPR team is tasked with managing the cardiac arrest (including CPR, drug administration and airway management), preparing for potential coronary angiography and assembling/priming the ECMO circuit (only in the on-demand group). If the patient achieves a return of spontaneous circulation (ROSC) or the ECPR team identifies contraindications for ECMO therapy (e.g., asystole, severe comorbidities or higher degree of lactic acidosis),^8^, ^9^ the ECMO circuit may not be utilized (Graphical abstract: options B and D). Conversely, if the patient remains in refractory cardiac arrest and meets ECMO eligibility criteria,^8^ ECPR can be considered in accordance with guideline recommendations (Graphical abstract: options A and C).

Analyses were conducted and retrospectively compared between the pre-primed and on-demand groups, involving 30 randomly selected adult patients who received ECPR treatment with VA-ECMO for therapy-refractory cardiac arrest (either in-hospital cardiac arrest [IHCA] or out-of-hospital cardiac arrest [OHCA]) at the tertiary care center between January 2020 and June 2023. A web-based randomization tool, specifically https://www.random.org, was employed to ensure unbiased selection. This process involved inputting the full list of eligible anonymized IDs of patients into the tool, which then generated a randomized selection of cases during the phase of new ECMO circuit assembly and the phase of pre-primed ECMO circuits. In instances where cardiac arrest occurred during an active procedure within the cardiac catheterization laboratory, the time interval between the onset of cardiac arrest and the initiation of ECMO flow was recorded. Conversely, if the cardiac arrest occurred outside of the cardiac catheterization laboratory (i.e., IHCA on ward or OHCA), the time difference (in minutes) between the patient entering the cardiac catheterization laboratory and the initiation of ECMO flow (“door to ECMO time”) was extracted for each case. The “door to ECMO times” between the pre-primed and on-demand groups were compared.

VA-ECMO cannula were inserted in the cardiac catheterization laboratory, with times to start of the ECMO circuit flow recorded by the cardiac catheterization laboratory nurses. Patients who received VA-ECMO treatment for reasons other than refractory cardiac arrest (such as cardiogenic shock) or outside the cardiac catheterization laboratory (such as in the intensive care unit [ICU] or emergency department) were excluded from the analyses. Two databases were accessed for hospital data: the intensive care electronic patient information system (COPRA System GmbH) for ICU patient data and Centricity™ RIS-i (GE Healthcare) for interventional data. The sample size of included patients was based on the availability of eligible patients who underwent ECPR during the study period. Due to the limited number of ECPR cases, a formal power calculation could not be performed. All retrieved data were manually verified by two independent and blinded investigators through meticulous review of discharge reports and cardiac catheterization laboratory protocols. The two independent investigators were blinded to the treatment group (pre-primed versus on-demand primed ECMO circuits) when verifying data related to time to ECMO initiation and patient outcomes.

### Assessment of psychological strain

To analyze the stress levels of the ECPR team during cases involving pre-assembled/pre-primed ECMO circuits (Graphical abstract: options A and B) versus newly assembled/primed ECMO circuits (Graphical abstract: options C and D), the Perceived Stress Questionnaire (PSQ-20) was utilized.^10^ The PSQ-20 is an established instrument for assessing stressful circumstances, encompassing four subscales: “worries,” “demands,” “tension” and “joy”, with a total of 20 items. This questionnaire has been validated in various adult patient cohorts.^11^ The overall score is calculated as the sum of each item. In collaboration with experienced psychologists, the PSQ-20 was adapted to the specific context of ECPR cases. The modified PSQ-20 was distributed to the whole ECPR team at the tertiary care center, which includes interventional cardiologists with fellowship training in intensive care medicine and/or emergency medicine, as well as experienced ICU and cardiac catheterization laboratory nurses, who have participated in at least 10 ECPR cases. The modified PSQ-20 was completed anonymously. ECPR providers indicated how often the presented statements applied to them on a 4-point Likert scale ranging from 1 (“almost never”) to 4 (“usually”) (Supplemental Table 1). The survey was prospectively conducted after the implementation of pre-primed ECMO circuits and all survey participants had prior experience with both methods.

### Statistical analysis

Data analyses were conducted using R Core Team 2020 (Vienna, Austria). Normality of the data was assessed using the Shapiro–Wilk test. Categorical and continuous variables were compared using Chi-square tests and Student’s t-tests for normally distributed variables or Fisher’s exact test and Wilcoxon-Mann-Whitney *U* test for non-normally distributed variables, respectively. Normally distributed continuous variables are presented as mean (standard deviation, SD), non-normally distributed variables as median (interquartile range, IQR), and categorical variables as frequency (percentage). A two-tailed P-value of less than 0.05 was considered statistically significant. The results for culture samples were reported using 95 % confidence intervals (CI), which were calculated based on binomial probability estimates.

## Results

### ECMO circuit sterility

In this study, 10 ECMO circuits were aseptically assembled and primed for 21 days. All sample cultures from these ECMO circuits remained negative for microbial and fungal growth throughout the 21-day period: Zero out of 120 (0 %, 95 % CI for binomial probability 0–0.03) positive cultures were observed. These results indicate the safety of pre-assembling, pre-priming and storing ECMO circuits for at least 21 days in the recovery room of the cardiac catheterization laboratory.

### Time to ECMO initiation

Fifteen patients were retrospectively, randomly selected for the pre-primed group (pre-assembled/pre-primed ECMO circuit) and fifteen for the on-demand group (newly assembled/primed ECMO circuit) by using a web-based randomization system. Patient characteristics were similar between both groups and are detailed in Table 1. The patients had a mean age of 63 (±11) years, with 25 (83 %) being male. Acute myocardial infarction was the most frequent cause of cardiac arrest (17 [57 %] cases). The mean time until initiation of ECMO flow was 13 (IQR 9–17) minutes in the pre-primed group and 31 (IQR 27–44) minutes in the on-demand group (P < 0.001) (Graphical abstract).

**Table 1:** Patient and treatment characteristics.

**Characteristics**	**Pre-assembled ECMO** **(n = 15)**	**Newly assembled ECMO** **(n = 15)**	**Total cohort** **(n = 30)**	**P value**
**Demographic data**				
Age (years)	63 (12)	63 (11)	63 (11)	0.912
Male sex	11 (73.3 %)	14 (93.3 %)	25 (83.3 %)	0.327
Body mass index (kg/m^2^)	28^25^, ^26^, ^27^, ^28^, ^29^, ^30^, ^31^, ^32^	28^24^, ^25^, ^26^, ^27^, ^28^, ^29^, ^30^, ^31^	28^25^, ^26^, ^27^, ^28^, ^29^, ^30^, ^31^, ^32^	0.492
History of peripheral artery disease	1 (6.7 %)	2 (13.3 %)	3 (10.0 %)	0.999

**Cardiac arrest data**				
OHCA	6 (40.0 %)	6 (40.0 %)	12 (40.0 %)	0.999
IHCA (during on-going procedure in the cardiac catheterization laboratory)	5 (33.3 %)	4 (26.7 %)	9 (30.0 %)	0.999
IHCA (on ward)	4 (26.7 %)	5 (33.3 %)	9 (30.0 %)	0.999
Endotracheal intubation in the cardiac catheterization laboratory	4 (26.7 %)	2 (13.3 %)	6 (20.0 %)	0.369

**Cause of cardiac arrest**				
Acute myocardial infarction	7 (46.7 %)	10 (66.7 %)	17 (56.7 %)	0.4
Pulmonary embolism	2 (13.3 %)	0	2 (6.7 %)	
Severe respiratory failure	1 (6.7 %)	2 (13.3 %)	3 (10.0 %)	
Acute heart failure	1 (6.7 %)	1 (6.7 %)	2 (6.7 %)	
Pericardial tamponade	1 (6.7 %)	0	1 (3.3 %)	
Severe atrial fibrillation	1 (6.7 %)	0	1 (3.3 %)	
Aortic dissection	1 (6.7 %)	0	1 (3.3 %)	
Severe aortic stenosis	0	1 (6.7 %)	1 (3.3 %)	
Coronary dissection	0	1 (6.7 %)	1 (3.3 %)	
Unknown	1 (6.7 %)	0	1 (3.3 %)	

**ECMO implantation**				
Off-hour shift*, weekend or public holiday	8 (53.3 %)	4 (26.7 %)	12 (40.0 %)	0.264
Time until ECMO initiation (minutes)**	13^9^, ^10^, ^11^, ^12^, ^13^, ^14^, ^15^, ^16^, ^17^	31^27^, ^28^, ^29^, ^30^, ^31^, ^32^	23.5 (13.9)	<0.001
**Outcome**				
In-hospital survival	4 (26.7 %)	2 (13.3 %)	6 (20.0 %)	0.648

Values are displayed as frequency (percent), mean (standard deviation) or median [interquartile range]. *Between 4.30PM and 8 AM during regular week days. **Time from entering the cardiac catheterization laboratory (if cardiac arrest occurred outside of the cardiac catheterization laboratory) or from onset of cardiac arrest (if cardiac arrest occurred during an on-going procedure in the cardiac catheterization laboratory). ECMO, extracorporeal membrane oxygenation; IHCA, in-hospital cardiac arrest; OHCA, out-of-hospital cardiac arrest.

### Psychological strain

A total of six ECPR physicians and 25 ECPR nurses (20 [80.0 %] working in the ICU and 5 [20.0 %] in the cardiac catheterization laboratory) were enrolled to complete the PSQ-20. Pre-assembled and pre-primed ECMO circuits were perceived to significantly reduce personal demands, stress, worries and frustration, while improving feelings of personal accomplishment and safety. However, the use of pre-primed ECMO circuits in ECPR cases did not lead to increased job satisfaction, higher physical energy levels or more personal time for both ECPR physicians and nurses.

**Fig. 1:**
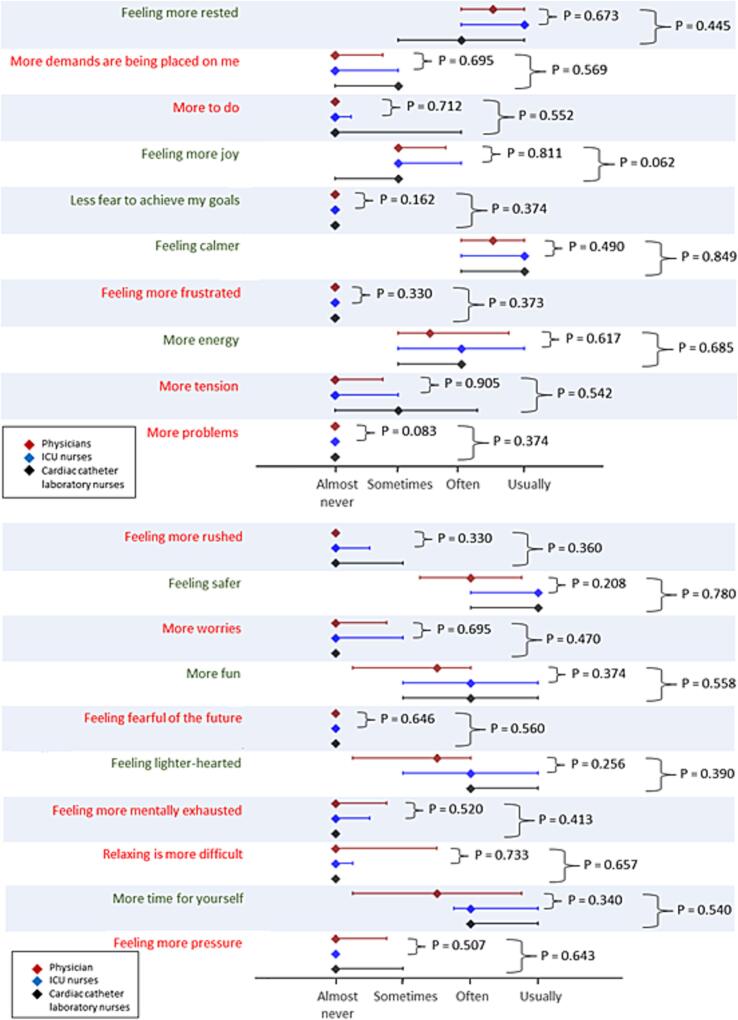
Modified Perceived Stress Questionnaire-20 (PSQ-20) answered by ECPR professionals. To evaluate the stress levels of the ECPR team when using aseptically pre-assembled/pre-primed versus newly assembled/primed ECMO circuits, the PSQ-20 was employed. This validated questionnaire assesses stress through subscales of “worries”, “demands”, “tension” and “joy” using 20 items. The PSQ-20 was modified for ECPR contexts and anonymously completed by experienced cardiologists and nurses, with responses recorded on a 4-point Likert scale. ECMO, extracorporeal membrane oxygenation; ECPR, extracorporeal cardio-pulmonary resuscitation; ICU, intensive care unit; PSQ-20, Perceived Stress Questionnaire.

ECPR physicians and nurses provided similar responses across all PSQ-20 items (each P > 0.05) (Fig. 1). The mean PSQ-20 score for ECPR physicians was 31.4 (±17.1; range = 13 – 60) and 34.3 (±10.9; range = 0 – 57) for ECPR nurses (p = 0.621). The Cronbach’s alpha for the PSQ-20 was 0.77, indicating acceptable internal consistency.

## Discussion

This study found that aseptically pre-assembled and pre-primed ECMO circuits maintained sterility for at least 21 days and significantly reduced the time to ECMO initiation compared to on-demand assembly. Additionally, using these pre-primed circuits decreased psychological strain on the ECPR team, contributing to a more efficient and less stressful emergency response.

### Real-world application

Notably, the ECMO machines remained sterile for at least 21 days in this study, confirming the safety of a pre-priming approach. The study authors did not aim for a longer study period, given the clinical experience that ECMO machines are utilized on average every 7–10 days at the tertiary care center. Another study demonstrated ECMO circuit sterility for up to 65 days, where the ECMO machines were stored in a separate supply room with a controlled temperature of approximately 22 °C.^12^ However, this study aimed to simulate actual real-world clinical practice, hence the ECMO machines were stored in the patient recovery room of the cardiac catheterization laboratory, potentially exposed to hospital staff and patients walking by. Despite these circumstances, the ECMO circuits remained free from microbial and fungal growth. Therefore, pre-assembled and pre-primed ECMO circuits could be stored in rooms used for daily clinical care, where they can be easily accessed during ECPR cases.

### Impact on ECMO initiation time

This study demonstrated that pre-assembly and pre-priming of ECMO machines significantly reduced the time until ECMO initiation. The interval between cardiac arrest and the onset of ECPR is a crucial predictor of patient survival.^13^, ^14^, ^15^ Therefore, pre-assembly and pre-priming of ECMO circuits could reduce mortality in this complex patient population by minimizing low-flow times. Depending on the experience of the ECPR team, assembling and priming ECMO circuits typically requires 10–20 min, while the ideal therapeutic window for ECPR initiation is proposed to be within 60 min of patient collapse.^16^ In a previous study conducted by our research group involving 1,014 ECPR cases across 32 medical centers, it was found that the average time required from the onset of cardiac arrest to the initiation of ECPR was approximately 54 min.^17^ This process could be further expedited with pre-assembled and pre-primed ECMO circuits. To further reduce low-flow times, several pre-hospital ECPR programs have been established to increase ECMO accessibility for patients with OHCA distant from ECPR centers. For instance, vehicle-based and helicopter-borne ECPR programs have been introduced in the Paris area.^18^, ^19^ Additionally, multiple case reports and series on pre-hospital ECPR have been published^18^, ^20^, ^21^ and several forthcoming pre-hospital ECPR trials are in progress.^22^, ^23^, ^24^ The findings of this study strongly suggest that utilizing pre-assembled and pre-primed ECMO machines can significantly reduce the time required for ECMO initiation. This approach is recommended to enhance the efficiency and responsiveness of ECMO procedures, particularly in critical situations such as cardiac arrest.

### Resource allocation and efficiency

Additionally, pre-assembled and pre-primed ECMO circuits allow for more efficient resource allocation. The ECPR team must assemble, prime and de-air the ECMO circuit using strictly aseptic techniques. During these time-consuming procedures, the responsible personnel are temporarily withdrawn from critical patient care tasks, such as continuous CPR, airway and drug management or preparing for emergency coronary angiography. This issue is particularly pronounced during evening and night shifts, as well as weekends, when hospital staff is limited and patient outcomes tend to be worse.^25^ Moreover, in certain cases, acutely assembled and primed ECMO circuits may be discarded due to the occurrence of ROSC or unmet ECPR eligibility criteria (Graphical abstract: option D), thereby unnecessarily diverting resources from more critical tasks. If not further required, these newly assembled/primed ECMO circuits might be disposed of due to potential contamination risks, as rapid ECMO assembly may not always adhere to strict sterility. Disposal of unused ECMO circuits also increases hospital expenses. Pre-assembled and pre-primed ECMO circuits can mitigate these issues by ensuring readiness while conserving valuable resources and potentially reducing unnecessary costs.

### Infection risk and prevention

At this tertiary care center, sepsis has occurred in approximately 14 % of ECPR cases,^26^ a rate comparable to the incidence of hospital-acquired infections during ECMO therapy reported in the medical literature.^27^ Device-related infections can be mitigated through preventive measures, such as pre-assembly and pre-priming of ECMO circuits under strictly aseptic techniques, as demonstrated in this study. The risk of contamination is significantly higher when ECMO circuits are assembled under time constraints (Graphical abstract: option C).^28^ Pre-assembled and pre-primed ECMO circuits, prepared under controlled sterile conditions, can therefore potentially help reduce the incidence of infections and improve patient outcomes.

### Psychological impact on ECPR teams

Apart from improved low-flow times, this study showed that the use of pre-primed ECMO machines resulted in a more settled inner feeling, reduced psychological tension, worries and insecurities, as well as more effective ICU shifts with better personal goal achievement of healthcare providers, indicating a clear preference for pre-assembled and pre-primed ECMO circuits. However, pre-primed ECMO circuits did not lead to more joyful work or higher physical energy levels, as ECPR cases are inherently medically challenging and complex. ECPR physicians and nurses provided similar responses across all items of the modified PSQ-20.

Almost all ICU admissions are unplanned emergencies requiring ICU professionals to rapidly adjust and treat patients with unclear outcomes.^29^ Numerous studies have shown that ICU professionals experience high levels of anxiety, moral distress, depression, burnout and posttraumatic stress disorder.^30^, ^31^ ECPR patients, in particular, present comprehensive and unstable medical cases involving multiple highly invasive and resource-consuming diagnostics and therapies within a short time period, including airway management, ECMO implantation, coronary angiography, renal replacement therapy, targeted temperature management, difficult transports and treatment of procedural complications.

### Models of ECMO care and professional well-being

Based on a study across 14 ECMO centers, it has been proposed that new nursing models must be considered for ECMO care, given the high intensity and complexity of ECMO patients.^32^ Notably, a recent study across five hospitals found that the feeling of personal accomplishment was significantly lower among ECMO professionals compared to non-ECMO professionals, while the burnout rate was similar between both groups.^31^ Although numerous studies focus on improving outcomes for ECMO patients and their relatives,^32^ there remains a considerable gap in assessing the psychological burden on ICU professionals treating ECMO patients. Given the results of this study, it is crucial to reduce the workload of ECPR wherever possible. Implementing pre-assembled and pre-primed ECMO machines can help conserve ICU resources and mitigate the psychological strain on healthcare professionals, thereby enhancing the overall efficiency and effectiveness of patient care.

## Limitations

There are certain limitations to this study. First, the study was conducted at a single tertiary care center, which may limit the generalizability of the findings. The ECMO circuit assembly and priming procedures might differ in other ECMO centers. However, to enhance the study's generalizability, the assembly and priming were performed by four independent individuals. Second, ECMO initiation times might also be influenced by the experience of the interventionalist and patient factors, such as obesity, pathology and morphology of femoral vessel access and the presence of peripheral artery disease. Nonetheless, ECPR cases were randomly selected for retrospective review, both before and after the intervention of ECMO circuit pre-priming, and the differences in ECMO initiation times were statistically significant. The observed reduction in ECMO cannulation times may have been influenced by increased provider proficiency over time, although the team composition of new and experienced providers remained comparable between both time phases. Third, the PSQ-20 was completed by a relatively small number of ICU physicians with extensive ECPR experience, who were part of the ECPR team at the tertiary care center. This small sample size could introduce potential bias. However, the responses of the physicians were consistent across all PSQ-20 items and closely matched those of the nurses, suggesting that the findings are robust despite the limited sample size.

## Conclusion

This study demonstrated that pre-assembled and pre-primed ECMO machines remain sterile for at least 21 days, shorten the time until ECMO initiation in patients with refractory cardiac arrest and considerably alleviate the psychological strain on the ECPR team (both physicians and nurses). Therefore, ECPR centers might consider pre-assembly and pre-priming of ECMO circuits to potentially improve outcomes for both ECPR patients and caregivers.

## Funding source

This research did not receive any specific grant from funding agencies in the public, commercial or not-for-profit sectors.

## CRediT authorship contribution statement

**Tharusan Thevathasan:** Writing – review & editing, Writing – original draft, Visualization, Validation, Supervision, Resources, Project administration, Methodology, Investigation, Formal analysis, Data curation, Conceptualization. **Sonia Lech:** Writing – review & editing, Methodology, Investigation, Formal analysis, Data curation. **Andreas Diefenbach:** Writing – review & editing, Resources, Methodology, Investigation, Data curation. **Elisa Bechthold:** Writing – review & editing, Validation, Methodology, Investigation, Formal analysis, Data curation. **Tim Gaßmann:** Writing – review & editing, Resources, Methodology, Investigation, Data curation. **Sebastian Fester:** Writing – review & editing, Resources, Methodology, Investigation, Data curation. **Georg Girke:** Writing – review & editing, Methodology, Investigation. **Wulf Knie:** Writing – review & editing, Methodology, Investigation. **Benjamin T. Lukusa:** Writing – review & editing, Methodology, Investigation, Data curation. **Sebastian Kühn:** Writing – review & editing, Resources, Methodology, Investigation, Data curation. **Steffen Desch:** Writing – review & editing, Methodology, Investigation, Data curation. **Ulf Landmesser:** Writing – review & editing, Supervision, Resources, Methodology, Investigation, Data curation. **Carsten Skurk:** Writing – review & editing, Writing – original draft, Validation, Supervision, Resources, Project administration, Methodology, Investigation, Data curation, Conceptualization.

## Declaration of competing interest

The authors declare that they have no known competing financial interests or personal relationships that could have appeared to influence the work reported in this paper.
