# Supplementary material for: Pre-assembled ECMO: Enhancing efficiency and reducing stress in refractory cardiac arrest care
Source: Resusc Plus. 2024 Oct 18;20:100800. doi: 10.1016/j.resplu.2024.100800 (PMC11513517; doi:10.1016/j.resplu.2024.100800)
Supplement: Supplementary Data 1 [file mmc1.docx]

**Supplemental Material**

| ***Supplemental Table 1.*** Perceived stress questionnaire (PSQ-20) | | | | |
| --- | --- | --- | --- | --- |
| Questions | Almost never | Sometimes | Often | Usually |
| 1. You feel more rested with an already established ECMO device. |  |  |  |  |
| 2. You feel like more demands are being placed on you with an already established ECMO device. |  |  |  |  |
| 3. You have more to do with an already established ECMO device. |  |  |  |  |
| 4. You feel like you are doing more things you really enjoy with an already established ECMO device. |  |  |  |  |
| 5. You fear being able to achieve your goals even less with an already established ECMO device. |  |  |  |  |
| 6. You feel calmer with an already established ECMO device. |  |  |  |  |
| 7. You feel more frustrated with an already established ECMO device. |  |  |  |  |
| 8. You have more energy with an already established ECMO device. |  |  |  |  |
| 9. You feel more tense with an already established ECMO device. |  |  |  |  |
| 10. Your problems seem to pile up more with an already established ECMO device. |  |  |  |  |
| 11. You feel more rushed with an already established ECMO device. |  |  |  |  |
| 12. You feel safer and more protected with an already established ECMO device. |  |  |  |  |
| 13. You have more worries with an already established ECMO device. |  |  |  |  |
| 14. You have more fun with an already established ECMO device. |  |  |  |  |
| 15. You are increasingly fearful of the future with an already established ECMO device. |  |  |  |  |
| 16. You feel lighter-hearted more often with an already established ECMO device. |  |  |  |  |
| 17. You feel more mentally exhausted with an already established ECMO device. |  |  |  |  |
| 18. You have more difficulty relaxing with an already established ECMO device. |  |  |  |  |
| 19. You have more time for yourself with an already established ECMO device. |  |  |  |  |
| 20. You feel more under pressure with an already established ECMO device. |  |  |  |  |
